# SPiNCAR: A systematic model to evaluate and guide actions for tackling AMR

**DOI:** 10.1371/journal.pone.0265010

**Published:** 2022-03-10

**Authors:** Giulia Bravo, Giovanni Cattani, Francesca Malacarne, Pierfrancesco Tricarico, Luca Arnoldo, Laura Brunelli, Carla Zotti, Maria Luisa Moro, Giuseppe Diegoli, Patrizio Pezzotti, Antonino Bella, Annalisa Pantosti, Ornella Punzo, Adriano Grossi, Martina Barchitta, Antonella Agodi, Greta Castellini, Matteo Marrazzo, Francesco Auxilia, Gianluca Cavallaro, Giovanni Loris Alborali, Elena Mazzolini, Silvio Brusaferro

**Affiliations:** 1 Department of Medicine, University of Udine, Udine, Italy; 2 Friuli Centrale Healthcare University Trust, Udine, Italy; 3 Giuliano Isontina Healthcare University Trust, Trieste, Italy; 4 Risk Management Unit, ULSS7 Pedemontana, Bassano del Grappa, Italy; 5 Department of Public Health and Pediatrics, University of Turin, Turin, Italy; 6 Regional Health and Social Agency of Emilia-Romagna, Bologna, Italy; 7 Direzione Generale Cura della Persona, Salute e Welfare Regione Emilia-Romagna, Bologna, Italy; 8 Italian National Institute of Health, Rome, Italy; 9 ASL Roma 1, Rome, Italy; 10 Department of Medical and Surgical Sciences and Advanced Technologies "GF Ingrassia", University of Catania, Catania, Italy; 11 Unit of Clinical Epidemiology, IRCCS Orthopedic Institute Galeazzi, Milan, Italy; 12 ASST Fatebenefratelli Sacco, Milan, Italy; 13 Department of Biomedical Sciences for Health, University of Milan—ASST Fatebenefratelli- Sacco, Milan, Italy; 14 Mit Studio, Rome, Italy; 15 Department of Epidemiology, Istituto Zooprofilattico Sperimentale delle Venezie, Padua, Italy; North Carolina State University College of Veterinary Medicine, UNITED STATES

## Abstract

**Background:**

Italy records very alarming levels antimicrobial resistance (AMR), so a National Action Plan on Antimicrobial resistance (PNCAR) was developed, adopting the AMR European Union’s recommendations based on the results of the ECDC site visit of January 2017. For achieving PNCAR objectives, it is necessary to support and harmonize the implementation of recommendations in all the different healthcare levels (regional authorities and local trusts), so the SPiNCAR project was launched to create a tool for reaching this goal.

**Methods:**

We developed a framework based on a scientific literature and national and international guidelines. Firstly, we identified the major intervention areas for tackling AMR, then, for each area, we built a set of standards, both for regional authorities than for local trusts. Every standard is composed by a set of essential and additional criteria, which refer to a minimum or supplemental performance level respectively. The contents were firstly discussed by the project’s team during face-to-face kick-off meetings, then confirmed with Delphi methodology and finally validated through a pilot study.

**Results:**

The final framework consists of seven different areas that reflect the PNCAR structure: Governance, Surveillance and Monitoring, Appropriate Use of antimicrobials, Healthcare-associated Infections (HAIs) control and prevention, Education and Training, Alliance among Stakeholders, Implementation. The total number of standards for the regional framework was 34 with 264 criteria and for the local version 36 criteria with 279 standards.

**Conclusion:**

The ongoing use of this tool, developed on international evidences and recommendations that were tailored on the Italian specific context, allows monitoring the improvement achieved over time and plan the next steps.

## Introduction

The most important national and international institutions recognize the increase of antimicrobial resistance (AMR) as a global threat [1–3]. In fact, excessive and inappropriate use of antimicrobial drugs is leading to an acceleration of the natural process of selection of microorganisms capable to defy the selective pressure of pharmacological compounds [4]. Data reported from the most recent surveys conducted by the European Centre for Disease Prevention and Control (ECDC) show that in acute Italian hospitals over 40% [4] of the patients are in treatment or prophylaxis with at least one antimicrobial agent, while in the long-term care facilities (LTCFs) the prevalence of residents with antimicrobial prescription was 5.5% [5]. Data related to the local primary care facilities and the residential and semi-residential structures would not be less worrying considering results of the annual report on drugs consumption published by the Italian Drugs Agency (AIFA) (OsMed Report) [6], where antimicrobials have been confirmed among the first categories of drugs for overall consumption. This situation worsened due to the massive antibiotics use in the livestock and agricultural sectors, with Italy ranking among the top countries of the European Union both for antimicrobial consumption and AMR levels [7–9]. Italy is one of the European Union countries with the higher presence of AMR [10], but, analyzing specifically the situation, a very heterogeneous context has emerged with wide difference in prevalence of AMR among the Regions for the majority of the strains selected as indicators [11]. AMR threatens the effective treatment of some previously curable infections, resulting in longer duration and severity of the course of diseases, undermining the safety of immunosuppressive treatments and surgery, increasing the cost of healthcare, and possibly leading to adverse outcomes such as disability and death [12]. It was estimated that in 2015 there were 671,689 infections due to resistant microorganisms in Europe (95% CI, 583,148–763,966), with over 33,000 attributable deaths and nearly 900,000 DALYs (Disability Adjusted Life-Years) [13]. These findings are even more alarming if we consider the current epidemiological prediction for 2,050 of about 10 million deaths attributable to infections caused by resistant microorganisms worldwide [14]. To properly contrast this evolution, the adoption of structured and integrated programs on AMR is recommended by the scientific literature and both national and international institutions [2, 15–17] and reiterated by the European Council in the *“Council Conclusions on the next steps under a One Health approach to combat antimicrobial resistance”* document [18]. An actual One Health approach, including human, veterinary/zootechnical and agricultural sectors interdisciplinary strategies carried out within and outside hospitals, is therefore essential to achieve the appropriate use of antimicrobials and the containment of the spread of antimicrobial resistance. Of note, in Italy each region is responsible for planning and organizing its health services, therefore the organization and implementation of health strategies and services may vary from region to region. The regionalization of health hampers a unified approach even in the AMR field, where adopting clear guidelines and monitoring strategies is of paramount importance.

In a context where Italy records very alarming levels for antimicrobial consumption and AMR prevalence, the National Action Plan on Antimicrobial resistance (PNCAR) 2017–2020 [19] was born, adopting the AMR European Union’s recommendations and planning actions based on the results of the ECDC site visit of January 2017 [20]. PNCAR aims to promote a common AMR approach at different local levels; in particular, an effective program to contrast AMR must act as a single and concrete response to multiple local needs, reducing as much as possible the spread of bacterial resistance both in the hospital and at the local level, promoting appropriate use of antimicrobials that guarantees an efficient, safe and high-quality level of care, fostering the collaboration between professionals in different fields and sectors, while enhancing their skills and distinctive characteristics.

Even if many standards and guidelines have been identified, they always focus on a single specific context, for example on the appropriateness of antimicrobial prescription in the hospital [21], or in the veterinary field [22, 23], or on hospital stewardship [24] or infection prevention and control [25], missing the opportunity to harmonize strategies to be adopted in a unitary One Health vision.

From this perspective, we developed a framework of standards built to facilitate the progressive implementation of a single national strategy and the planning of actions to tackle AMR at different levels (regional and local), in accordance with PNCAR goals. The structure of the framework derives from a previous self-assessment tool developed to measure healthcare institutions performance on Clinical Risk Management (CRM) to guide improvements over time (CARMINA) [26]. Its structure was fit to the framework we were looking for, and conceived as a tool able to adapt to different contexts and to foster a continuous improvement in the organization.

The present study aims to describe the development process and validation of the standards’ framework.

## Methods

### The framework development

The framework was developed based on a scientific literature review and an analysis of national and international guidelines and regulations available on AMR and infection prevention and control (IPC). Firstly, different intervention areas were identified considering the most important field of action to tackling AMR. For each area a set of quality standards, both for regional and local level implementation, were identified, underlining the core elements to achieve the PNCAR goals. Every standard is then composed by a set of essential and additional criteria, which refer to a minimum or supplemental performance level respectively. The different criteria reflect actions to be improved, ideally arranged in an increasing complexity model that firstly addresses basic issues and then deals with more advanced problems. This model allows not only to define the achieved level and the room for improvement but at the same time provides a road map for the future.

The contents were, at first, modified by the project’s team during a face-to-face kick-off meeting, and then updated several times using the Delphi technique to reach a consensus [27, 28]: a panel of experts (not involved in the framework development process) was asked to provide its contribution, improving the tool’s contents based on their specific experiences. The panel was composed of Italian experts in the field of AMR and IPC having different professional profiles and coming from different sectors (doctors, vets, policy-makers, public health researchers, academics). Experts’ identity and anonymity were preserved during the subsequent online Delphi consensus procedure rounds to ensure impartiality.

The methodology was explained to all experts involved. They were invited to evaluate each standard and criterion using a nine-point Likert scale based on two aspects: feasibility and relevance. In particular, during the evaluation process, they were asked to consider how much the criteria could be applied to the context, and if they would have been useful to achieve the PNCAR goals. Using the 9-point Likert Scale rating method, the experts assigned a rating equal to 1 when they considered an item useless or impossible to detect and 9 when they found it completely satisfactory.

At each Delphi round, conducted both at the regional and local level, a median score was calculated for each criterion. Considering the quantitative results and the accompanying notes of experts, the framework was improved and then returned to each expert for the following Delphi round until a final consensus was achieved. The obtained scores were used to select the criteria.

At the end of the consensus procedure, the agreement among experts was calculated through Kendall’s W index, for both areas and standards respectively. The more the W tends to 1, the greater the agreement between the evaluators.

### The framework validation

After the final consensus obtained with the Delphi technique, two pilot studies were arranged to validate the framework. Two convenience samples of Italian regions and healthcare trusts were enrolled, using representatives from the north, center and south of Italy, to ensure the compliance with the current heterogeneous experiences observed throughout the country. Each region and healthcare institution enrolled was requested to identify one person or more who, due to the type of role played and experience in the AMR field, could conduct a self-assessment of their own structure. The compilers were sent a web-based survey, built with Survey Monkey Inc. (San Mateo, California, USA) containing the framework, also equipped with instructions and explanatory notes helping to clarify and guide the filling in. Compilers were asked to test the framework by compiling it as far as their region/healthcare trust was concerned, using the options “yes”, “no” or “not applicable”; they were also given the possibility to add comments when needed. Comments collected helped highlight critical issues at the regional and local level. After the web-survey completion, an adapted version and face validated System Usability Scale (SUS) Questionnaire was administered to each compiler to assess the subjective usability of the framework.

## Results

### Framework structure and Delphi consensus of expert

A total of three rounds of Delphi consensus process were conducted to evaluate the initial structure of the framework and to obtain the consensus of experts. In the first step, the structure and contents of the framework were evaluated in a face-to-face meeting by 54 experts divided by human and veterinary area and by topic. The other two Delphi rounds were conducted online and involved 15 and 12 experts for the regional level and 12 and 11 for local one, respectively. In each rounds the selection of experts was made respecting the geographical representation.

At each Delphi round, separately at the regional and local level, a median score was assessed for each criterion. Furthermore, for some criteria, it was not possible to calculate the agreement indicator due to a large number of missing values.

The tool was therefore updated based on the quantitative results obtained and the various comments reported by the evaluators and was sent back to each expert for the subsequent review cycle.

At the end of the consensus procedure, an indicator of concordance by area between the experts was also calculated (W by Kendall): overall, the agreement index varies from 0.4 to 0.8.

Following the evaluation of the experts’ agreement, the structure was reshaped in order to consider the qualitative observations about structure, terminology, and contents.

The final framework consists of seven different *Areas*, chosen to reflect the PNCAR structure: *Governance*, *Surveillance and Monitoring*, *Appropriate Use of antimicrobials*, *Healthcare-associated Infections (HAIs) control and prevention*, *Education and Training*, *Alliance among Stakeholders*, *Implementation*.

Each area includes a different number of *standards* (ranging from two to ten) and each standard a different number of *criteria* (ranging from 15 to 89).

The *standards* address the core issues of each specific *area*. Following the One Health approach, whenever possible we addressed the *area*, not only from a medical point of view, but also through criteria related to all the categories involved.

To allow comparison over time, it was necessary to move from self-assessment to an objective and quantitative assessment; for this reason, a score was assigned for each *criterion*, based on the scores assigned by the experts during the last Delphi round.

Only when all the essential criteria are met, the score referred to the additional criteria can also be added to the total. In the end, the framework compilation will return a total score for each standard and each area.

### Framework—Final version

The final version of the framework is depicted in Table 1.

**Table 1:** Number of *standards* and *criteria* for each *Area* in the framework.

	Regional		Local	
Area	*Standard*	*Criteria*	*Standard*	*Criteria*
Governance	3	31	4	33
Surveillance and Monitoring	10	89	7	75
Antimicrobial Appropriate Use	6	45	6	48
Healthcare-associated Infections Control and Prevention	2	22	3	28
Education and Training	4	29	5	32
Alliance (Public awareness)	7	33	6	28
Implementation	2	15	5	35

The first *area*, *Governance*, requires a formal identification of targets, priorities, and responsibilities. This *area* evaluates the design and planning for the management of appropriate use of antimicrobials, contrasting resistance -even in the veterinary field- and surveillance, prevention, and control of HAIs.

The *Surveillance and Monitoring Area* evaluates the ability of the system to monitor the consumption of antimicrobials in humans and animals, promptly identifying alert microorganisms, track resistance to antimicrobials and HAIs, and to report the monitored data.

The *area Antimicrobial Appropriate Use* explores the implemented strategies to limit the incorrect use of antimicrobials in humans, farms, and pets, for example by recommending the use of guidelines in prescribing treatment in specific settings, or by introducing regional policies.

In the *Healthcare-associated Infections Control and Prevention area*, the criteria investigate the presence of procedures and programs related to healthcare associated infections (HAIs)–first of all, proper hand hygiene—highlighting the need for multimodal strategies.

The *Education and Training area* has the purpose to evaluate the actions done in the field of staff education, encouraging the realization of a dedicated course for all healthcare workers, with particular attention to new hires; specific courses addressed to veterinarians, dentists, pharmacists are also recommended. In addition, specific skills are required for managers and dedicated teams working in AMR and IPC programs.

The *area Alliance* addresses the tricky challenge of active involvement of the civil society, by information campaigns and knowledge dissemination initiatives. Moreover, attempts and strategies to involve health and veterinary staff working in the private sector are also included.

The last *area Implementation* helps monitoring the ability of the system to evaluate the impact that AMR and HAIs programs have gained on a yearly basis.

### The pilot study and the usability of the framework

Seven regions were available for the pilot study: Veneto, Lombardy, and Piedmont for the North-East and North-West respectively; Lazio and Umbria for the central regions; Calabria and Sicily for the South. A total of 15 compilers tested the regional framework, seven of them (47%) also providing a completed SUS as well. A good level of regional framework usability emerged from the results (SUS score: median value = 75).

Then, 11 experts coming from the aforementioned regions took part in the healthcare trusts pilot study. 91% of these completed the entire survey, also filling the usability questionnaire. The score obtained was 68 (median value, indicating a Good Rating).

## Discussion and practical implications

### Framework development method

The framework is conceived as a cyclical process, able to adapt to different contexts, and continuously improve the actions taken. It has been developed in several steps, in order to create a tool adapted to the Italian context but, at the same time, able to summarize and channel the international evidence on the topic. Starting from a literature review and a synthesis of guidelines and tools provided by international organizations, the first group of criteria was collected based on the main findings in the field of contrast to AMR. The next step was finding a consensus among the project experts in the discussion, the choice and organization of the criteria, leading to the first draft of the framework, designed with the contributions of different viewpoints and experiences.

The face-to-face meeting and then the two Delphi consensus rounds, with the involvement of several experts from hospitals, veterinarians, national organizations, and academic institutions contributed, allowed us to refine the framework, improving it with additions, cuts, appropriate redefinitions, and also to find a consensus on the priority criteria and the attribution of a score on each one. The pilot studies (regional and local) provided us with information on the feasibility of compilation, the difficulties in interpreting the criteria, and the reliability of the questionnaire.

This strategy–although complex in its multi-step design—has allowed to identify and to develop a validated and flexible framework in a specific field lacking clear gold standards.

### Tool benefits

From the pilot studies conducted, the framework has proved to be quite flexible in describing very different organizations, and we retrieved a good level of satisfaction from the compilers: this result is remarkable because the tool is intended to be operative and useful for policymakers to develop, implement, evaluate and monitor over time an AMR program within a One Health approach.

In addition, to allow comparison over time of the progress of the own organization, this questionnaire allows benchmarking between similar structures through time: if the first comparison helps to identify strengths and weaknesses—and consequently where to direct further efforts—the second allows to identify "good practices" and learn from examples available in similar contexts.

### Limitations

The framework was created as a national monitoring instrument for the achievement of the goals specified by the PNCAR, even if it was not always possible to identify standards and criteria for each objective, sometimes because they were too generic, sometimes due to lack of scientific evidence on what to evaluate and on how to do it–e.g. in the environmental sector.

The final framework presents a total of 264/279 *criteria*, covering the seven *areas*: it means that filling the questionnaire requires a considerable amount of time and the involvement of several staff members or stakeholders in order to answer to specific standards and to obtain a global and accurate view of the local and regional status.

Other criticalities are linked to the great regional variability of the actions in progress on AMR, with the difficulty of having a unitary picture of the situation; the project, therefore, aims to pursue this goal, with the objective of standardizing the activities and identifying common strategies.

As a result of the complex heterogeneity existing between Italian Regions and health organizations—of different sizes, geographical locations, economic and human resources dedicated to the program- we expect a large variation in the scores of the different realities. This certainly highlights the criticalities of the Italian context and could discourage the most disadvantaged organizations from exposing their weaknesses and being compared to better-performing organizations at the national level. Nevertheless, this process is also a necessary step to understand the status quo and define a plan to tackle AMR adapted to a specific scenario, hopefully also involving the central government to invest in the weakest links in the chain.

Finally, the creation of users’ guidelines, also based on feedback from the pilot studies, should allow greater homogeneity in the interpretation of the criteria, but the possibility of different perceptions in the evaluation still remains.

## Conclusions and recommendations

Tackling AMR is urgently needed, but it requires a systematic approach to respond to the complex and dynamic nature of the issue. Several organizations worldwide are working to identify standards and criteria to evaluate and guide the actions undertaken by different countries [29, 30], but, to our knowledge, this is the first comprehensive framework that investigates the different areas of AMR in several sectors and at different levels.

Designed to guide and facilitate Italian organizations in the path towards the construction and implementation of the PNCAR goals, the framework allows a transparent national monitoring system with the possibility of comparing similar organizations (i.e. benchmark).

The ongoing use of the tool allows monitoring the improvement achieved over time and plan the next steps. The high number of criteria, if on the one hand represents a limit to the compilation, on the other allows having a sufficiently in-depth analysis of the actions taken. At the same time, highlighting the minimum levels for each standard may guide the organizations in choosing the order of priority of intervention.

Although drawn up on the basis of international evidence and recommendations, the framework was developed with great attention to the Italian specific reality and therefore represents a tailor-made tool. Nevertheless, the whole method could be adapted to other countries after a few necessary adjustments, cuts, and additions.

The next phases of the project include the creation of a web-based platform to facilitate the compilation, a graphic return of the results, a users’ guideline to the correct use of the framework, and its sharing with the organizations involved through structured and dedicated site visits.
